# Efficient Chlorostannate Modification of Magnetite Nanoparticles for Their Biofunctionalization

**DOI:** 10.3390/ma17020349

**Published:** 2024-01-10

**Authors:** Maria O. Zolotova, Sergey L. Znoyko, Alexey V. Orlov, Petr I. Nikitin, Artem V. Sinolits

**Affiliations:** 1 Prokhorov General Physics Institute of the Russian Academy of Sciences, 38 Vavilov Street, 119991 Moscow, Russiaalexey.orlov@kapella.gpi.ru (A.V.O.); 2 National Research Nuclear University MEPhI (Moscow Engineering Physics Institute), 31 Kashirskoe Shosse, 115409 Moscow, Russia; 3 Vernadsky Institute of Geochemistry and Analytical Chemistry, Russian Academy of Sciences, Kosygin Str. 19, 119991 Moscow, Russia

**Keywords:** magnetite nanoparticles, magnetometry, biofunctional coating, stannous chloride, antibody conjugate, magnetic immunochromatography, drug delivery, nanobioconjugation, in vitro diagnostics, magnetic separation, biomolecule conjugation, targeted therapeutics, biofunctional nanomaterials, molecular imaging

## Abstract

Magnetite nanoparticles (MNPs) are highly favored materials for a wide range of applications, from smart composite materials and biosensors to targeted drug delivery. These multifunctional applications typically require the biofunctional coating of MNPs that involves various conjugation techniques to form stable MNP–biomolecule complexes. In this study, a cost-effective method is developed for the chlorostannate modification of MNP surfaces that provides efficient one-step conjugation with biomolecules. The proposed method was validated using MNPs obtained via an optimized co-precipitation technique that included the use of degassed water, argon atmosphere, and the pre-filtering of FeCl_2_ and FeCl_3_ solutions followed by MNP surface modification using stannous chloride. The resulting chlorostannated nanoparticles were comprehensively characterized, and their efficiency was compared with both carboxylate-modified and unmodified MNPs. The biorecognition performance of MNPs was verified via magnetic immunochromatography. Mouse monoclonal antibodies to folic acid served as model biomolecules conjugated with the MNP to produce nanobioconjugates, while folic acid–gelatin conjugates were immobilized on the test lines of immunochromatography lateral flow test strips. The specific trapping of the obtained nanobioconjugates via antibody–antigen interactions was registered via the highly sensitive magnetic particle quantification technique. The developed chlorostannate modification of MNPs is a versatile, rapid, and convenient tool for creating multifunctional nanobioconjugates with applications that span in vitro diagnostics, magnetic separation, and potential in vivo uses.

## 1. Introduction

Magnetite nanoparticles (MNPs) represent a favorable material for a wide range of multifunctional applications including magnetic labeling for molecular and cellular studies [1,2,3], magnetic immunoassays [4], magnetic separation techniques in biotechnology [5,6,7], targeted drug delivery systems [8], and contrasting in medical in vivo imaging [9,10], etc. MNPs are synthesized via a wide variety of methods using different iron precursors and reagents to produce nanoparticles of various shapes, such as cubic [11] or rod-shaped [12], sizes, and magnetic and other properties [1]. Among the most common methods is co-precipitation, which is relatively easy to use for producing MNPs with good superparamagnetic parameters suitable for biomedical and biosensor applications [2]. It is known that as the magnetite nanoparticle size decreases, the magnetization behavior of the particle enormously decreases [13]. Magnetic signals of MNPs can be measured using the magnetic particle quantification (MPQ) technique [4,10], superconducting quantum interference devices (SQUIDs) [14], magnetic force microscopy [15], and vibrating sample magnetometry (VSM) [16], etc. VSM was used to characterize the magnetic properties of MNPs synthesized in this work as it is a more versatile and cost-effective technique and does not require cryo-temperatures as SQUID does, and it is capable of detecting ultra-small magnetic moments in contrast to magnetic force microscopy.

The MNP surface lacks functional groups capable of forming strong covalent complexes with biomolecules. Therefore, various approaches were developed for the functionalization of the MNP surface to enable covalent conjugation with biomolecules [3,5,6,8,10]. An initial modification of the MNP surface with silica or silica-like coatings is often used to introduce hydroxyl or organic groups for further functionalization [9]. One of the most common approaches for the covalent grafting of biomolecules to MNPs involves treating the MNP surface with (aminopropyl)-triethoxysilane [7,17] to introduce amine functional groups. These amine groups can then be further terminated with carboxyl groups through interactions with succinic anhydride, creating a pathway for the covalent grafting of biomolecules through carbodiimide linking [18,19,20,21]. Covalent complexes between MNPs and biomolecules are robust and can maintain their composition in different media, although the release of biomolecules from these complexes may be impeded [22].

The use of certain organic solvents or linking substances for covalently grafting biomolecules to the MNP surface may lead to biomolecule deactivation or toxicity in the biomolecule–nanoparticle complex. Therefore, non-covalent approaches for binding biomolecules to MNPs are also under consideration. Typically, MNPs are initially modified with polymers or hydrogels to facilitate non-covalent bonds with biomolecules or drugs [23]. In such cases, non-covalent interactions between the capped nanoparticles and biomolecules or drugs are explored [24,25]. The non-covalent approach for forming complexes between biomolecules and nanoparticles can result in a reduced risk of protein denaturation or drug deactivation, is generally simpler than the covalent approach and offers lower toxicity of the complex [26]. The simplest example involves non-covalent interactions between a protein and unmodified nanoparticles. However, in this scenario, the load of the protein/drug onto the nanoparticle may be quite low, as might the strength of the substance retention on the nanoparticle surface.

Here, we propose to modify the MNP surface with Sn compounds to enhance the non-covalent strength of biomolecule retention on the nanoparticle surface. It is widely recognized that Sn forms a strong bond with a S atom, resulting in the formation of very sparingly soluble sulphides [27]. Moreover, Sn can form complexes with sulphur-containing amino acids. As an example, Sn(II) interacts with cysteine to produce a complex, in which Sn(II) is bound with S (thiol group), O (carboxyl group) and N (amino group) [28]. One may consider chlorostannated inorganic materials for the immobilization of thiol- and disulphide-containing biomolecules, e.g., cysteine-containing proteins [29].

To the best of our knowledge, R.A. Messing was the first to report on the application of chlorostannated ceramic titan oxide scaffolds for the immobilization of the enzyme urease [30]. This application led to an increase in the enzyme retention on the scaffold without significant changes in catalytic activity. Subsequently, SnCl_2_ was proposed for the modification of glass surfaces [31]. More recently, chlorostannated porous silica was used for the covalent immobilization of lipase [32]. However, despite the common functionalization of MNPs with tin oxides [33,34], methods for obtaining chlorostannated MNPs that could be promising for immobilizing biomolecules on them are still to be developed.

In this work, we have developed a cost-effective method for the chlorostannate modification of MNPs that provides efficient one-step conjugation of biomolecules. We have validated this method using MNPs prepared via optimized co-precipitation that involves degassed water, argon atmosphere, and pre-filtered FeCl_2_ and FeCl_3_ solutions. Stannous chloride was employed to modify the MNP surface. We have performed a comprehensive characterization and comparison of the obtained chlorostannated MNPs with both carboxylate-modified and unmodified MNPs.

The performance of the obtained functional MNPs was successfully demonstrated by the biorecognition of folic acid (FA) in the magnetic immunochromatography (IC) lateral flow format [4,10]. It should be noted that FA is of great practical importance in biomedicine, since it is often used as a recognition antigen for binding with the membrane of cancer cells for targeted drug delivery [35,36], and it is a vital vitamin for humans, which should be monitored during many diseases [37,38], etc. In our setup, mouse monoclonal antibodies to FA were used to create nanobioconjugates with the MNP. Additionally, FA–gelatin conjugates were immobilized on the test line of a nitrocellulose IC strip, and the specific trapping of the resulting nanobioconjugates via antibody–antigen interactions was registered via the highly sensitive magnetic particle quantification (MPQ) technique [4,10].

## 2. Materials and Methods

### 2.1. Materials

FeCl_3_·6H_2_O, FeCl_2_·4H_2_O, (aminopropyl)-triethoxysilane (APTES), succinic anhydride, bovine serum albumin (BSA) and 1-ethyl-3-(3-dimethylaminopropyl)carbodiimide (EDC) were sourced from Sigma-Aldrich (Burlington, MA, USA); NaOH, sodium citrate (trisubstituted, dihydrate), SnCl_2_·2H_2_O, dimethylformamide (DMF), acetone, NH_3_ (25% solution), trimethylamine and tetraethoxyortosilane (TEOS) were of analytical grade and were sourced from Chimmed (Moscow, Russia); ethanol (95%) was from Ferein (Moscow, Russia); phosphate buffer saline (PBS) (pH 7.4) was from PanEco Ltd. (Moscow, Russia); deionized water and Ar were of the highest grade. Monoclonal antibodies to folic acid (mAb-FA) (4.7 mg/mL, suspension) were purchased from the Research Center of Molecular Diagnostics and Therapy (Moscow, Russia), and their high specificity was checked by both the manufacturer and the authors in previous studies [37].

### 2.2. Degassing of Water

We used freshly degassed water to prevent the oxidation of Fe(II) by dissolved oxygen. Water degassing was carried out by boiling 350 mL of water in a 500 mL round-bottomed flask with magnetic stirring under reflux for 30 min. The hot, degassed water was put in dark place in a tightly capped vessel under Ar atmosphere, cooled to room temperature, and used within 1 day since degassing. All the solutions prepared using the degassed water were exposed to air for no longer than 15 min.

### 2.3. Magnetite Nanoparticles Synthesis

FeCl_3_·6H_2_O (6.000 mmol, 1.623 g) and FeCl_2_·4H_2_O (3.000 mmol, 0.600 g) were dissolved in 50 mL of degassed water forming a very slightly opalescent orange solution. The solution was filtered through a 0.2 μm filter under vacuum to separate slightly soluble oxides, hydroxides, and hydroxy- and oxychlorides, which occurred in FeCl_3_·6H_2_O and FeCl_2_·4H_2_O while in storage and might interfere the synthesis process as large crystallization centers. The filter color changed, and the solution became transparent. This indicated that FeCl_3_·6H_2_O and FeCl_2_·4H_2_O did contain slightly soluble components. The solution of FeCl_3_ and FeCl_2_ was placed in a three-necked flask and degassed under vacuum using a waterjet pump for 30 min.

NaOH (48.000 mmol, 1.920 g) was dissolved in 100 mL of degassed water, and the solution was placed into a dropping funnel connected to the three-necked flask with the solution of iron chlorides. The NaOH solution was gradually added to the solution of iron chlorides for 60 min, with the reaction mixture kept under Ar flow (<1 L/min) with non-magnetic stirring for 1 h (1300 rpm) using an overhead stirrer with a glass three-wing impeller to prevent the impact of strong magnetic fields on the forming MNPs. All subsequent synthesis stages were carried out under Ar flow, and one synthesis was performed under air. As the NaOH solution was added, the formation of a black suspension was observed, with the solution turning colorless. After all NaOH was added, the formed black suspension was heated to 90 °C using a water bath and stirred non-magnetically for 1 h. Then, the reaction mixture was cooled down to room temperature while stirring.

Sodium citrate dihydrate (7.350 g) was dissolved in 49.1 mL of degassed water to form a 50 mL 0.5M solution. MNPs were separated magnetically, and the supernatant was decanted. The magnetic nanoparticles were washed 3 times with degassed water with the use of a magnet to sediment them. After this stage, some aliquot of the MNP suspension could be collected to use pure MNPs.

The washed MNPs were resuspended in a sodium citrate solution, forming a highly stable suspension. The mixture was then heated up to 80 °C in a water bath under stirring. MNPs were sedimented with 50–100 mL of acetone and collected with a magnet. The supernatant was decanted. MNPs were washed with acetone one more time and dried under vacuum for 6 h. MNPs were resuspended in 50 mL of water, resulting in a suspension with a concentration of ~20 mg/mL if no aliquot of non-stabilized MNPs was collected.

The MNP suspensions were kept in tightly closed plastic tubes sealed with parafilm or glass vessels under Ar at room temperature. The citrate-stabilized (MNP-cit) suspension was stable for at least two weeks.

### 2.4. Functionalization of MNP with Amino Groups (MNP-NH_2_)

The MNP-cit were initially modified with silica via the hydrolysis of TEOS for better and further deposition of APTES. In a plastic 1.5 mL tube, 0.110 mL of the MNP-cit suspension was mixed with 0.100 mL of degassed water, 0.634 mL of ethanol, and 0.016 mL of NH_3_ solution and sonicated for 30 min in an ultrasonic bath. Then, 0.110 mL of TEOS was mixed with 0.018 mL of ethanol and added to the MNP suspension. The mixture was vigorously shaken for 3 h. MNP-SiO_2_ was sedimented via centrifugation (13,400 rpm, 15 min) and washed 3 times with ethanol and 3 times with degassed water.

The sedimented and washed MNP-SiOH suspension was mixed with 0.221 mL of degassed water, 0.634 mL of ethanol, and an NH_3_ solution of 0/0.05/0.4% in a plastic 1.5 mL tube and sonicated for 30 min in an ultrasonic bath. Then, 0.128 mL of 0/0.42/2% APTES ethanol solution was added to the MNP suspension. The mixture was vigorously shaken for 12 h. MNP-NH_2_ was collected magnetically and washed with ethanol 3 times and with degassed water 3 times.

### 2.5. Functionalization of MNP-NH_2_ with Carboxyl Groups (MNP-COOH)

A total of 33 mg (0.33 mmol) of succinic anhydride was dissolved in 1 mL of DMF with 0.014 mL of trimethylamine. MNP-NH_2_ was resuspended in the succinic anhydride solution, sonicated, and vigorously shaken for 2 h. MNP-COOH was sedimented via centrifugation (13,400 rpm, 15 min) and washed 3 times with DMF and 5 times with degassed water. MNP-COOH was resuspended in 0.315 mL of water to form a suspension of ~7 mg/mL.

### 2.6. Covalent Grafting of mAb-FA to MNP-COOH

A total of 0.007 mL of MNP-COOH was mixed with 0.043 mL of water. A series of samples were prepared by adding 2, 4, 8, and 0.016 mL of the diluted MNP-COOH suspension to 0.030 mL of 10% mass of EDC solution, then sonicated and washed with water once after 30 min. Subsequently, 0.002 mL of mAb-FA suspension was added to MNP-COOH-EDC, sonicated and incubated at 25 °C for 2 h.

### 2.7. Functionalization of MNP with SnCl

**Figure 1:**
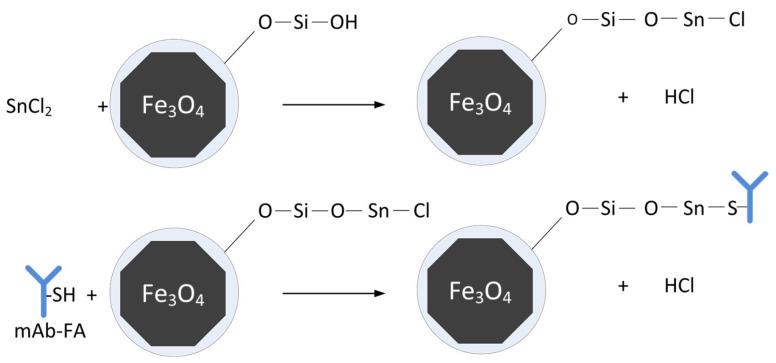
The proposed mechanism of protein binding with chlorostannated MNP (Y is a symbol of an antibody mAb-FA).

The proposed scheme of functionalization and conjugation with proteins is presented in Figure 1. In the first stage, the silica-coated MNP-SiO_2_ is modified with tin chloride. Then, the protein interacts with the chlorostannated surface via SH-containing groups, with the release of HCl forming a strong non-covalent complex.

We functionalized MNP-cit with SnCl_2_ in two different media leading to different results. Firstly, MNP-cit was functionalized in SnCl_2_ aqueous solution according to R.A. Messing’s work [30] and, secondly, in SnCl_2_ solution in DMF to prevent SnCl_2_ hydrolysis in solution, but not on the surface of MNPs.

In both methods, 5.65 mg (0.025 mmol) of SnCl_2_·2H_2_O was dissolved in 1 mL of the respective solvent (either water or DMF). A total of 0.110 mL of MNP-cit suspension was added to the SnCl_2_ solution, and the mixture was shaken for 1 h. Subsequently, the nanoparticles were sedimented with a magnet, washed (either with the same solvent or with water, depending on the solvent used), and finally resuspended in 0.315 mL of water to form a suspension with a concentration of ~7 mg/mL. The MNPs modified in DMF after washing in DMF were washed in water.

### 2.8. Non-Covalent Grafting of mAb-FA to MNP-X

A total of 0.007 mL of MNP-X (where X represents either SnCl_2_ or no modificator) was mixed with 0.043 mL of water. A series of samples were prepared by adding 0.002, 0.004, 0.008, or 0.016 mL of the diluted MNP-X suspension to 0.100 mL of water and 0.002 mL of the MAB-FAmAb-FA suspension, sonicated and incubated at 25 °C for 2 h.

### 2.9. Study of mAb-FA Conjugation with MNP

A total of 0.002 mL of MNP-X-mAb-FA (where X represents different types of MNP modification: COOH-EDC, SnCl_2_, or no modificator) from different series was added to 0.040 mL of 10% BSA in PBS (pH 7.4) for blocking the unreacted EDC, as was conducted in [39], and sonicated. Then, an IC test strip with a test line containing a conjugate of folic acid with gelatin was put into the MNP-X-mAb-FA suspension, kept for 15–30 min till all the liquid phase was absorbed, and dried. The magnetic signal distribution along the lateral flow strip was measured using an MPQ reader in a scanning mode similarly to the previously described procedure [40].

### 2.10. Sample Preparation for X-ray Diffraction Measurements and Vibrating Sample Magnetometry

MNPs were separated with a magnet from the MNP aqueous suspension, washed with acetone, and dried under vacuum using a waterjet pump for 6 h to get black-colored magnetic powder.

### 2.11. X-ray Diffraction Measurements

X-ray diffraction (XRD) measurements were carried out with Bruker D8 Advance at CuKα λ = 1.54060 Å at 2θ, rate 10–75°, step time t = 1.6 s, rotation 20 rpm in background-free cuvettes. PDF-2 rel. 2011 database was used for reference. Powder samples were obtained from aqueous suspensions via vacuum drying and kept in argon atmosphere until the measurements were obtained. The sample masses were ~200 mg.

### 2.12. Transmission Electron Microscopy (TEM)

TEM analysis was performed on a JEOL JEM-1400 (Akishima, Tokyo, Japan) microscope (120 kV). The aqueous suspension of MNP-cit (Ar) was dropped onto the surface of a formvar-coated copper grid (300 mesh), and the solvent was evaporated.

### 2.13. Vibrating Sample Magnetometry

The magnetic properties of the MNPs were investigated with a vibrating sample magnetometer Lakeshore 7407 (Lakeshore, Westerville, OH, USA) at room temperature. The samples were fixed onto a quartz holder with PTFE tape. The holder signal was subtracted from the experimental data. The samples amounts were 5.7 mg (MNP (powder)) and 5.1 mg (MNP-cit (powder)). The saturation magnetization values were assessed based on an approximation of the paraprocess region of the hysteresis loop to zero values of the magnetic field. The remanent magnetization and coercive field were determined as crosses of the hysteresis loop with Y and X axes, respectively. Data were processed with use of OriginPro 2018 software (version SR1, b9.5.1.195).

### 2.14. Magnetic Particle Quantification

In this study, we used the ultrasensitive magnetic particle quantification (MPQ) method for the quantitative detection of magnetic particles [4,10]. MPQ measures the nonlinear response of superparamagnetic nanomaterials at various combinatorial frequencies [4]. This method is characterized by outstanding analytical characteristics, including a remarkable limit of detection (i.e., 60 zeptomoles for 200 nm spherical magnetic particles or 87 particles for the detection of disk-shaped particles) and a linear range spanning seven orders of magnitude [40]. MPQ has demonstrated its efficacy in a wide range of in vitro [39,40] and in vivo applications [41], as well as for monitoring the biosynthesis of well-calibrated superparamagnetic nanoparticles using genetically encoded cells [42].

### 2.15. Colloidal Properties

Particle sizes and ζ-potentials of aqueous suspensions of MNPs with a concentration of ~1 mg/mL were measured using a ZetaSizer Nano ZS (Malvern, UK) at the following parameters: refractive index (RI) = 2.36 and absorption = 0.147. Aqueous suspensions of MNPs were washed with water in order to provide nearly the same, neutral pH for ζ-potential measurements.

## 3. Results and Discussion

### 3.1. MNP Characterization

#### 3.1.1. XRD Study of MNP

**Figure 2:**
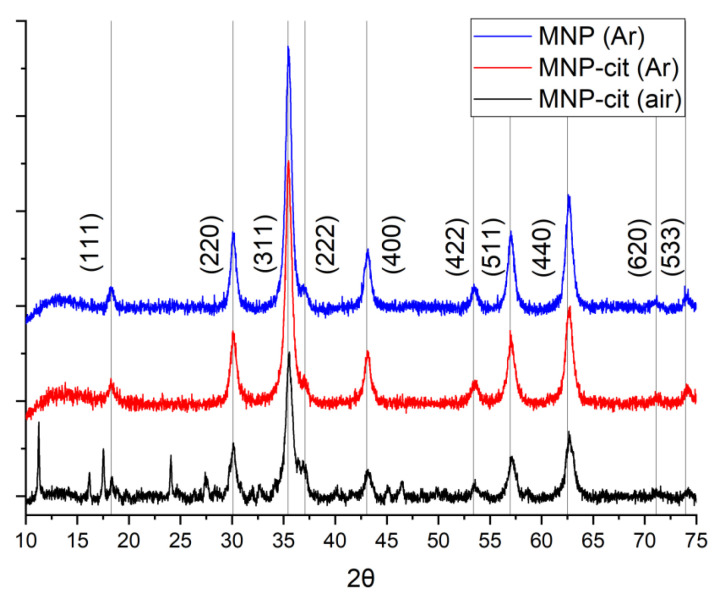
XRD of synthesized MNP. Gray lines correspond to magnetite according to PDF-2 rel. 2011 database.

The XRD analysis, as shown in Figure 2, confirms the composition of the synthesized MNPs as primarily magnetite. All 2θ values corresponding to magnetite align precisely with the data from the reference database (International Centre for Diffraction Data). Notably, the samples synthesized under argon (Ar) atmosphere exhibit excellent crystallinity with well-defined magnetite peaks, showcasing the high quality of these particles. In the XRD pattern of MNP-cit (air), some additional peaks corresponding to sodium citrate are observed. This suggests that this specific sample might have retained some sodium citrate due to incomplete washing, resulting in the formation of sodium citrate crystals during drying. Conversely, the XRD patterns of MNPs synthesized under Ar atmosphere do not exhibit sodium citrate peaks, indicating thorough washing. The different colloidal properties of these samples will be discussed further.

Therefore, we can infer the presence of a citrate shell on the surface of MNP-cit (Ar), although in small quantities. While there are indications of possible hematite, goethite, or lepidocrocite peaks in the MNP-cit (air) diffractogram, these peaks coincide with those of sodium citrate, making it challenging to confirm their presence definitively. Importantly, for the samples synthesized under an Ar atmosphere, only magnetite phases are observed.

The Scherrer equation [43], as follows, may be used to calculate the size of nano-scale crystallites in a solid sample with use of XRD data (1):

(1) $$\tau =\frac{K\lambda }{\beta \cos\theta }$$

where:

τ—the mean size of the crystalline domains;

K—dimensionless shape factor, which is usually equal to 0.93;

λ—X-ray wavelength;

β—broadening of the line at half the maximum intensity (FWHM);

θ—Bragg angle [43].

**Figure 3:**
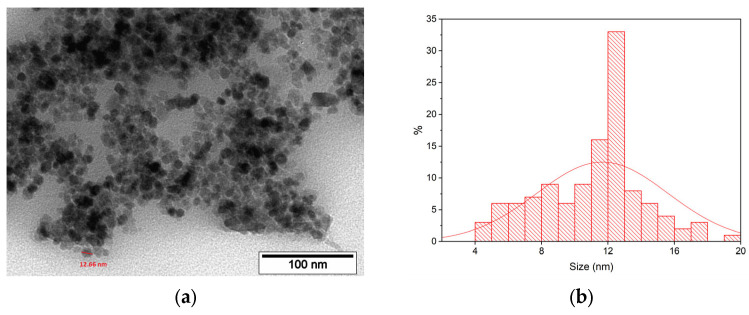
TEM microphotographs of synthesized MNP-cit (Ar) (**a**); size distribution of MNP-cit (Ar) according to TEM (**b**).

Crystallite sizes, according to the Scherrer Equation (1), are presented in Table 1. Regardless of the synthesis route presented in this study, the crystallite size of the MNPs remains consistently around 12 nm which is close to the size values according to TEM (Figure 3).

#### 3.1.2. TEM Study of MNP

Transmission electron microscopy of the as-synthesized MNPs indicates that MNPs are of a spherical or multifaceted shape with a median size of 12.36 nm (N = 100) which is close to the size values according to Scherrer’s equation (Table 1).

#### 3.1.3. Magnetization Measurements of MNPs

**Figure 4:**
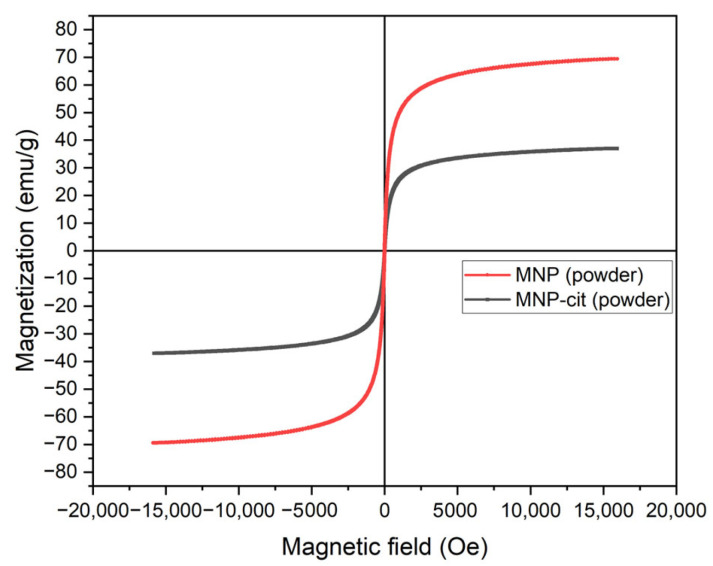
Dependence of magnetization of MNP powders on magnetic field.

According to vibrating sample magnetometry data, MNPs synthesized under Ar atmosphere demonstrate superparamagnetic behavior. The magnetization of MNP powder (Figure 4) is approximately twice as high as that of MNP-cit. This difference in magnetization between the MNP powder and the MNP-cit powder may be attributed to the fact that magnetization is calculated in reference to the mass of the sample, and MNP-cit are covered by citrate which may result in a bigger mass of the dried particles, so the specific magnetization parameter is lower for the MNP-cit powder.

**Figure 5:**
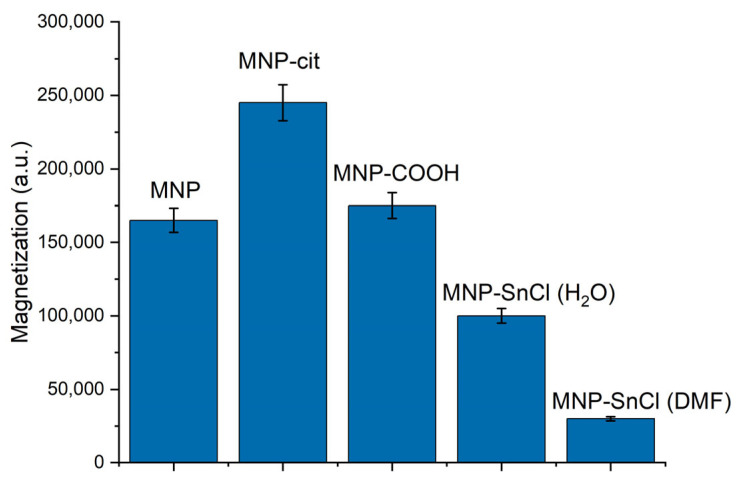
Comparison of magnetic signals from the synthesized MNP measured via an MPQ reader.

Magnetic signals of the synthesized and modified MNPs were measured via the MPQ technique (Figure 5). The presented magnetic signals are normalized to equal masses of the nanoparticles. Interestingly, the citrate-stabilized MNP-cit exhibited the highest signal, surpassing that of the pure MNPs. This difference may be attributed to the fact that the stabilized suspension contains nanoparticle agglomerates of smaller diameters than the non-stabilized particles. In contrast, the carboxylated MNP-COOH displayed a magnetic signal magnitude between that of the pure MNPs and MNP-cit. This indicates more efficient deagglomeration of MNP-COOH. This aspect will be explored in more detail below. Overall, our findings suggest that the modification of MNPs leads to a reduction in their magnetic signal, possibly due to an increase in size and mass.

#### 3.1.4. Colloidal Properties of As-Synthesized and Citrate-Stabilized MNP

**Figure 6:**
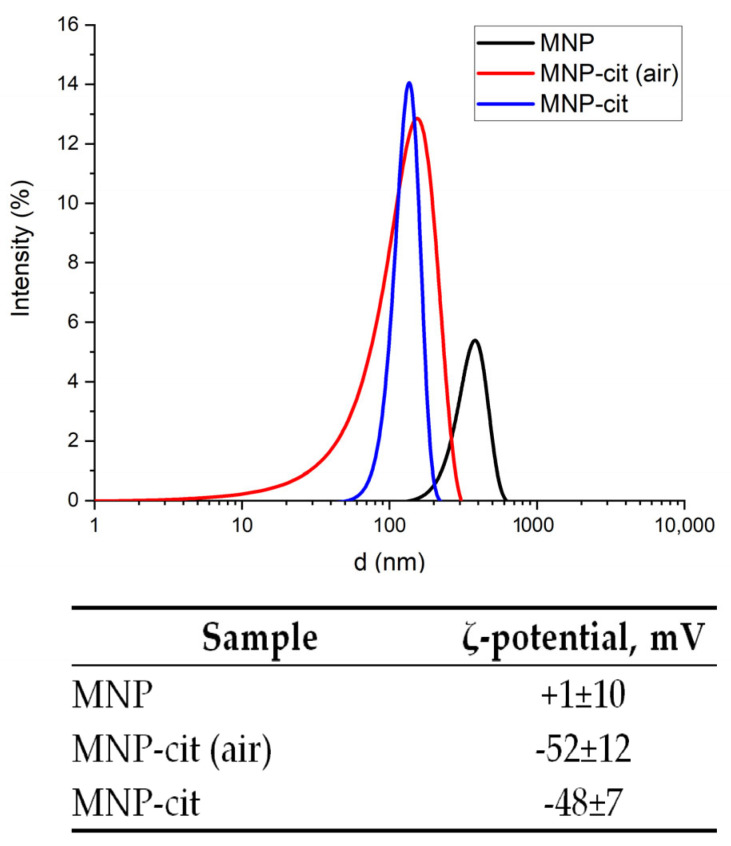
Particle size distribution and ζ-potential of the non-functionalized MNP.

As seen from the size distribution (Figure 6) and from SEM study (Figure S1), the mean size of the as-synthesized MNPs is about 380 nm. The MNP modification with sodium citrate significantly decreases the particle size down to 154 nm (for MNP-cit synthesized under air) and 136 nm (for MNP-cit synthesized under Ar). This, along with the crystallite size according to Scherrer’s equation and TEM (12 nm), indicates that particles are agglomerated in aqueous suspensions. The bigger particle size of non-modified MNPs may be explained by the nearly neutral surface charge of the particles (Figure 6), which leads to particle agglomeration in polar media such as water. The smaller size of citrate-stabilized MNP may be due to the deagglomeration of the nanoparticles because of the electrostatic repulsion of the negatively charged citrate on the MNP surface, which is confirmed by the relatively high negative ζ-potential.

#### 3.1.5. Colloidal Properties of MNP-SiO_2_

**Figure 7:**
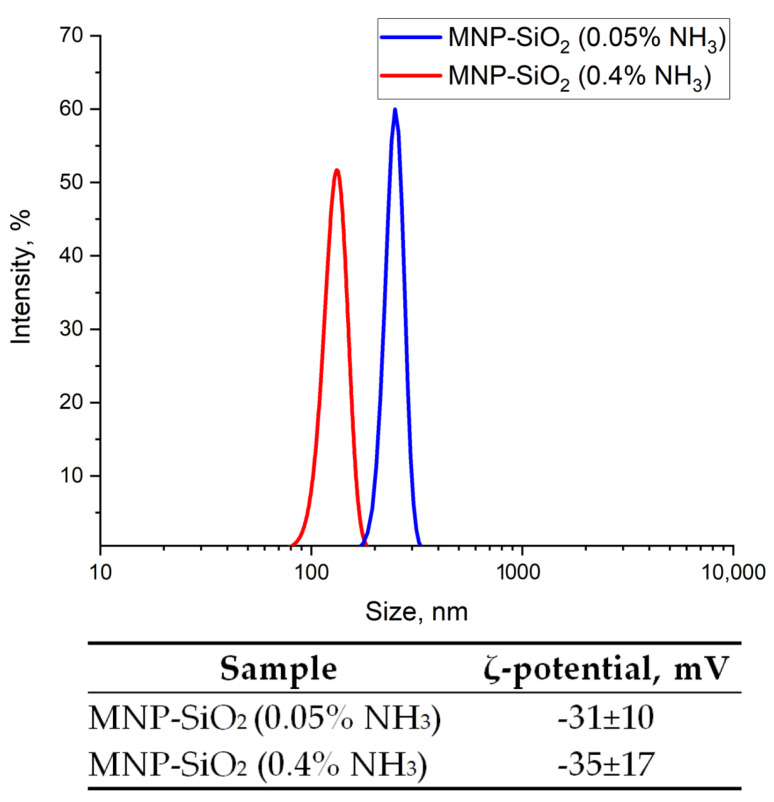
Effect of NH_3_ concentration on the particle size distribution and ζ-potential of MNP-SiO_2_.

An increase in NH_3_ concentration in the modification solution of TEOS from 0.05% to 0.4% leads to a decrease in the particle size of MNP-SiO_2_, from 250 nm to 146 nm (Figure 7). This indicates the deagglomeration of the modified MNPs, probably due to the higher hydrophilicity of silanol groups formed on the MNP surface and the presence of citrate, which may have remained in the solution. It is noteworthy that the particle size of MNP-SiO_2_ is larger than MNP-cit. This may suggest the formation of a silanol-containing layer, which increases the particle size.

#### 3.1.6. Colloidal Properties of MNP-NH_2_

**Figure 8:**
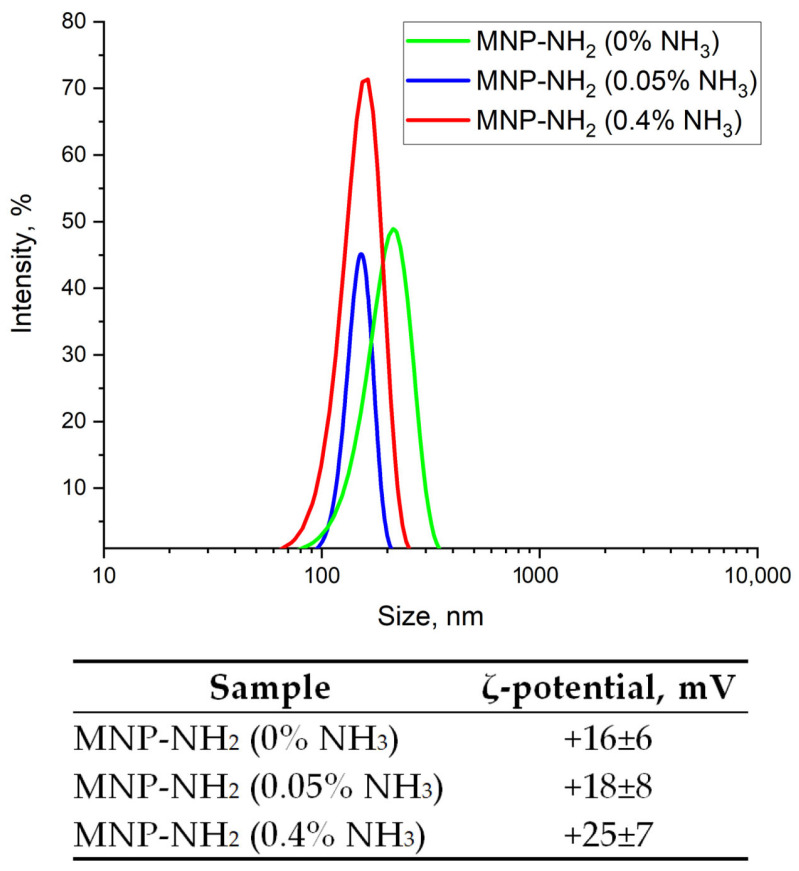
Effect of NH_3_ concentration on particle size distribution and ζ-potential of the aminated MNP.

The amination of MNP-SiO_2_ with APTES in solutions of different concentrations of NH_3_ has shown that the particle size decreases from 213 nm to 151 nm with an increase in the NH_3_ amount (Figure 8). This suggests the improved deagglomeration of MNPs in the presence of NH_3_ or the higher hydrolysis rate of APTES due to a higher pH. The ζ-potential of MNP-NH_2_ is positive, indicating the successful MNP functionalization with amino groups that are positively charged in an aqueous solution when dissociated. A growth in NH_3_ concentration leads to the rise in ζ-potential, from +16 mV to +25 mV, which may point toward the better modification of MNP-SiO_2_ with APTES due to the higher hydrolysis rate of APTES.

#### 3.1.7. Colloidal Properties of MNP-NH_2_ and MNP-COOH

**Figure 9:**
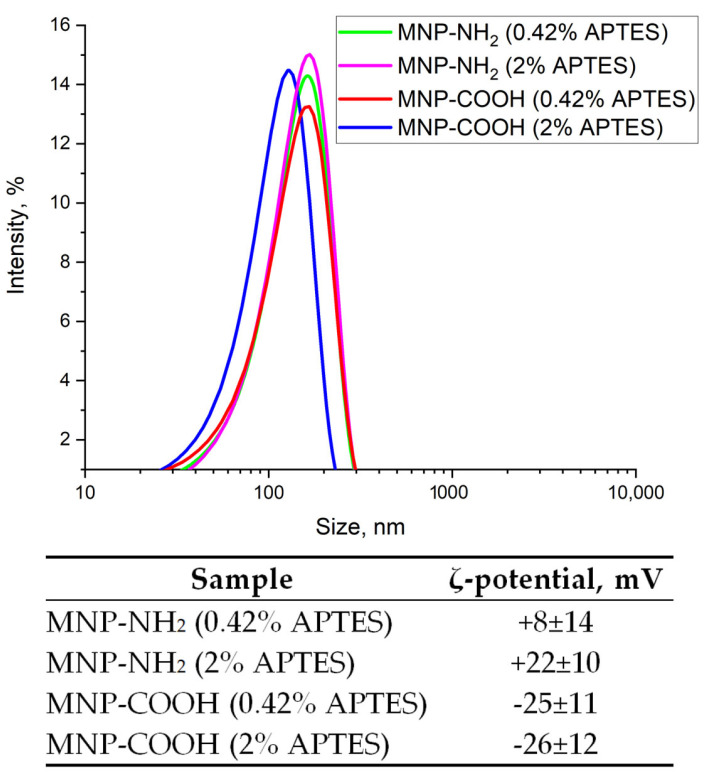
Effect of APTES amounts on particle size distribution and ζ-potential of the aminated and carboxylated MNPs.

Further modification of MNPs with APTES was performed with 0.4% NH_3_ with a varying amount of APTES (Figure 9). It was found that larger amounts of APTES produced MNP-NH_2_ with higher positive ζ-potential. This may indicate better modification of MNPs with APTES. In parallel with the increase in ζ-potential, the particle size of MNP-NH_2_ slightly reduced from 166 nm to 162 nm under growing APTES amounts. The carboxylation of amino groups of MNP-NH_2_ obtained at higher concentrations of APTES resulted in a particles size decrease from 163 nm to 128 nm pointing toward a more uniform coverage of the MNP-NH_2_ surface with amino groups at higher concentrations of APTES. The MNP-COOH size is smaller than that of MNP-NH_2_, probably due to the higher absolute value of the ζ-potential with a negative sign. Namely, the ζ-potential of MNP-COOH is about −25 mV, after amination at both concentrations of APTES, which indicates the successful carboxylation of MNP-NH_2_.

### 3.2. Magnetic Immunochromatography Studies of MNP Conjugation with Antibodies

#### 3.2.1. Conjugation of Pristine MNPs with mAb-FA

**Figure 10:**
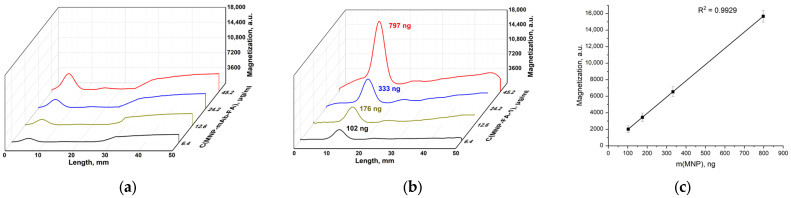
Distribution of magnetic signals along the strips registered using an MPQ device for pristine MNPs (**a**) and MNP-mAb-FA interacting with FA–gelatin conjugate (the indicated masses of MNPs correspond to the maximum binding on the test lines) (**b**); magnetic signal at the peak corresponding to MNPs binding on the test line against the mass of the involved MNPs (**c**).

Non-modified pristine MNPs were incubated with antibodies specific to folic acid. The non-covalent complexes MNP-mAb-FA were passed in a buffer solution through an IC lateral flow strip with FA–gelatin conjugates deposited on its test line. The main peaks on the magnetic signal distribution along the strip (Figure 10b) were observed at the test line containing FA–gelatin conjugates. The peaks observed are well pronounced. The magnetic signal linearly increases with the growth in the MNP quantity with good correlation (Figure 10c). One may see a plateau between 20 and 45 mm rising with the increase in the MNP concentration. The binding of pristine MNPs (without antibodies) with FA–gelatin conjugates is not observed (Figure 10a): small peaks near the start correspond to the start zone, and the plateau after 25 mm corresponds to the MNPs passed through the strip. These data indicate the weak binding of MNPs with antibodies conjugated to folic acid, even without any special surface modification of the nanoparticles.

#### 3.2.2. Conjugation of MNP-COOH with mAb-FA

**Figure 11:**
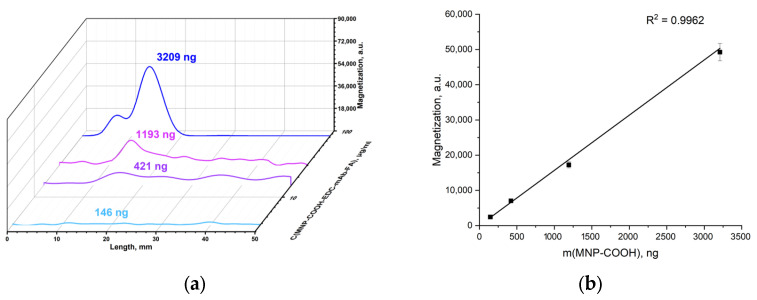
Distribution of magnetic signals along the IC strips measured using an MPQ device for MNP-COOH-EDC-mAb-FA interacting with FA–gelatin conjugate (the indicated masses of MNPs correspond to the maximum binding on the test lines) (**a**); magnetic signal at the peak corresponding to MNPs binding on the test line against the mass of MNPs involved (**b**).

MNP-COOH was modified with an EDC linker to produce MNP-COOH-EDC for the covalent conjugation with the mAb-FA antibody. Covalent complexes are stronger than non-covalent ones, and their stability in different media is supposed to be higher. This makes them more suitable for different assays. The covalent complexes MNP-COOH-EDC-mAb-FA were passed through IC strips with the FA–gelatin conjugates deposited on their test lines. The main peaks on the signal distribution (Figure 11a) are observed at about 12 mm similarly to the experiments with MNP-mAb-FA. The peaks are well pronounced, and the magnetic signal linearly increases under growth in the MNP quantity with good correlation (Figure 11b). The intensity of the magnetic signal rises with an increase in MNP-COOH-EDC-mAb-FA concentration. The quantity of MNP-COOH-EDC-mAb-FA bound to the FA–gelatin conjugate is much higher than that of MNP-mAb-FA. This indicates better functionalization of the former complexes than the latter. So, the covalent process allows for the binding of a larger amount of antibodies with MNP-COOH through an EDC linker than in the case of pristine MNPs.

#### 3.2.3. Conjugation of MNP-SnCl(H_2_O) with mAb-FA

**Figure 12:**
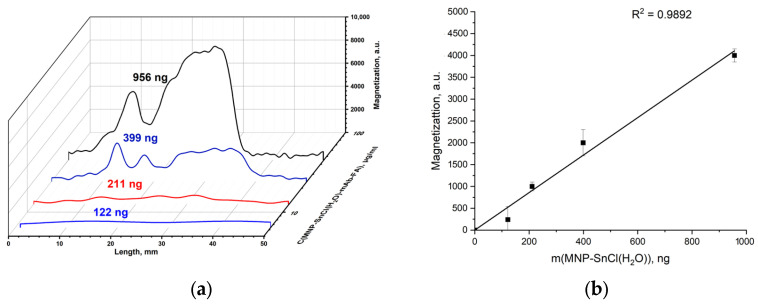
Distribution of magnetic signals along the IC strips measured using an MPQ device with MNP-SnCl(H_2_O)-mAb-FA against FA–gelatin conjugate with masses of MNPs at the peak corresponding to binding (**a**); magnetic signal at the peak corresponding to nanoparticle binding on the test line against the mass of MNPs involved (**b**).

The chlorostannated-in-water MNP-SnCl(H_2_O) was conjugated with the antibody mAb-FA and passed through the IC strip with the FA–gelatin conjugate deposited on its test line. According to the magnetometry data (Figure 12a), MNP-SnCl(H_2_O)-mAb-FA can bind with the FA–gelatin conjugate. This confirms that MNP-SnCl(H_2_O) are functionalized with the mAb-FA antibody. The amount of MNP-SnCl(H_2_O)-mAb-FA bound to the FA–gelatin conjugate is between that of the pristine MNPs and MNP-COOH. This indicates that the binding of mAb-FA to MNP-SnCl(H_2_O) is stronger than that to MNPs and weaker than that to MNP-COOH. There is a big shoulder on the signal distribution (Figure 11a) right after the main peak, which suggests that a large amount of nanoparticles are not associated with FA–gelatin conjugates. The signal dependence on the nanoparticle concentration is linear (Figure 12b). The use of chlorostannated MNPs modified with SnCl_2_ in aqueous media presents a challenge in terms of achieving the optimal nanoparticle–antibody complex binding to FA–gelatin conjugates. Possible factors influencing this issue include the potential hydrolysis of SnCl_2_ and the presence of Sn–Cl functional groups on the MNP surface when exposed to aqueous environments.

#### 3.2.4. Conjugation of MNP-SnCl(DMF) with mAb-FA

**Figure 13:**
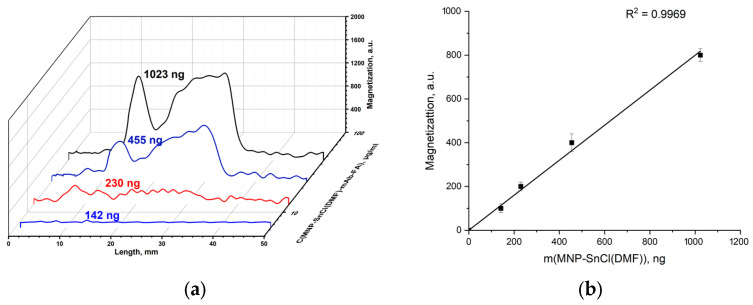
Distribution of magnetic signals along the IC strips registered using an MPQ device with MNP-SnCl(DMF)-mAb-FA against FA–gelatin conjugates with masses of MNP at the peak corresponding to binding on the test line (**a**); magnetic signal at the peak corresponding to nanoparticle binding on the test line against the mass of MNPs involved (**b**).

The chlorostannated-in-DMF-solution MNP-SnCl(DMF) was conjugated with the mAb-FA antibody and passed through the IC strip with FA–gelatin conjugates deposited on its test line, as in the abovementioned experiments. As can be seen from the signal distribution (Figure 13a), the magnetic signal is lower than that from MNP-SnCl(H_2_O)-mAb-FA, but the amount of bound particles is higher than in the previous case. This indicates a higher binding of antibodies within the MNP-SnCl(DMF)-mAb-FA complex. Furthermore, the peak corresponding to the shoulder is lower. The magnetic signal linearly depends on the MNP-SnCl(DMF)-mAb-FA mass (Figure 13b). Altogether, these facts suggest better binding of the mAb-FA antibody with MNP-SnCl(DMF)-mAb-FA, possibly due to the lower hydrolysis rate of SnCl_2_ in DMF.

It should be noted that the developed magnetic IC metrology could be useful for the investigation of many other functionalized magnetic nanoparticles, which are extensively studied, for example, for targeted hyperthermia treatments of cancer [44], the isolation and identification of circulating tumor cells [45], the diagnosis and treatment of cardiovascular diseases [46], and magnetic systems for personalized and precision medicine [47], etc. In addition, studies like those discussed above on the interaction of FAconjugated nanoparticles with other bio and chemical objects are of great interest for various applications, such as the development of targeted cancer contrast agents for MRI [48] or biosensors for preventive medicine [49], the adsorption of unfavorable chemical compounds and biological pollutants [50], and many others.

## 4. Conclusions

In this work, we proposed a novel easy-to-use, rapid and cost-effective technique for MNP functionalization with tin chloride. The chlorostannated MNPs exhibited superior non-covalent binding capabilities for proteins compared to their pristine counterparts. The rate of the modification of chlorostannated MNP with protein was estimated using an MPQ-based magnetic immunochromatographic lateral flow assay. The modification of the MNP surface with SnCl_2_ led to the formation of chlorostannated nanoparticles capable of binding with monoclonal antibodies. The relative amounts of mAb-FA bound to magnetic nanoparticles was found to be in the following sequence: MNP (pristine) < MNP-SnCl < MNP-COOH. While the antibody binding to pristine MNPs is non-covalent, it is covalent (via an EDC linker) for MNP-COOH. Therefore, the antibody interaction with MNP-SnCl is stronger than the non-covalent bond and weaker than the covalent one. Furthermore, we have found that the MNP modification by SnCl_2_ in DMF is more preferable than in H_2_O, possibly due to the lower hydrolysis rate of SnCl_2_ in aqueous media both in solution and on the surface of MNPs. The magnetometric immunochromatography can be applied for the rapid quantification of interactions between modified or non-modified MNPs with various biochemical agents. The chlorostannated MNP surfaces exhibit great potential for conjugation with biomolecules due to the formation of strong Sn–S bonds. These surfaces can accommodate larger quantities of biomolecules compared to pristine MNPs. The chlorostannated MNPs are promising for multifunctional applications in magnetic immunoassays, drug delivery systems, and the magnetic separation of biomolecules. Further investigation is required to fully explore and harness their potential in these fields. An easier release of biomolecules from chlorostannated MNP surfaces that are non-covalently bonded may be the more desirable option for drug delivery than from MNP-COOH that bonds with biomolecules covalently—this is to be studied in future research. Conjugates of mAb-FA with chlorostannated MNP may serve as sensing material for the detection of FA in solutions, and a more thorough investigation is needed.

## Figures and Tables

**Table 1:** Crystallite sizes of MNP according to the Scherrer equation.

	MNP (Ar)	MNP-Cit (Ar)	MNP (Air)
d, nm	12.32	11.93	11.72

## Data Availability

Data are contained within the article.

## Supplementary Materials

The following supporting information can be downloaded at: https://www.mdpi.com/article/10.3390/ma17020349/s1, Figure S1: SEM-microphotographs of MNP-cit (a); EDX spectra of MNP-cit (b).
