# Supplementary material for: Efficient Chlorostannate Modification of Magnetite Nanoparticles for Their Biofunctionalization
Source: Materials (Basel). 2024 Jan 10;17(2):349. doi: 10.3390/ma17020349 (PMC10820483; doi:10.3390/ma17020349)
Supplement: Supplementary file 1 [file materials-17-00349-s001.zip › materials-2732245-supplementary.pdf]

Supplementary Materials

According to TEM microphotographs and calculations with Scherrer's equation (provided in original manuscript), crystallites sizes are about 12 nm. Agglomerates of MNP with sizes 50-100 nm are observed at SEM-microphotographs (Figure S1,a). The conducted EDX (Figure S1,b) confirmed that the synthesized citrate-stabilized MNP contain Fe, O and C, Na that have come from sodium citrate during MNP modification.

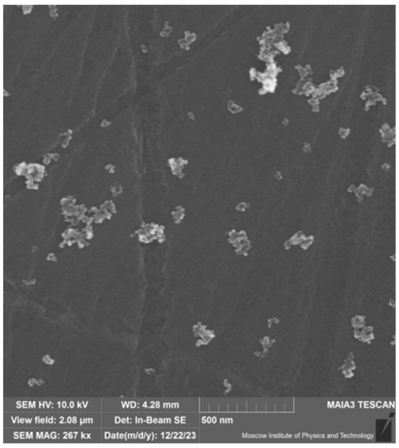

(a)

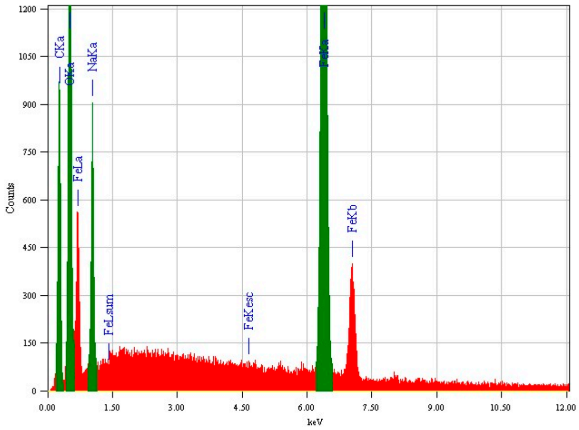

(b)

Figure S1. SEM-microphotographs of MNP-cit (a); EDX spectra of MNP-cit (b).
